# Hyperspectral Estimation of Canopy Leaf Biomass Phenotype per Ground Area Using a Continuous Wavelet Analysis in Wheat

**DOI:** 10.3389/fpls.2018.01360

**Published:** 2018-09-25

**Authors:** Xia Yao, Haiyang Si, Tao Cheng, Min Jia, Qi Chen, YongChao Tian, Yan Zhu, Weixing Cao, Chaoyan Chen, Jiayu Cai, Rongrong Gao

**Affiliations:** ^1^National Engineering and Technology Center for Information Agriculture, Key Laboratory for Crop System Analysis and Decision Making, Ministry of Agriculture, Jiangsu Key Laboratory for Information Agriculture, Nanjing Agricultural University, Nanjing, China; ^2^Department of Geography and Environment, University of Hawai‘i at Mānoa, Honolulu, HI, United States

**Keywords:** phenotypic parameter, canopy leaf biomass, continuous wavelet transform, optimal wavelet features, hyperspectral reflectance, wheat

## Abstract

To extend agricultural productivity by knowledge-based breeding and tailoring varieties to adapt to specific environmental conditions, it is imperative to improve our ability to acquire the dynamic changes of the crop’s phenotype under field conditions. Canopy leaf biomass (CLB) per ground area is one of the key crop phenotypic parameters in plant breeding. The most promising technique for effectively monitoring CLB is the hyperspectral vegetation index (VI). However, VI-based empirical models are limited by their poor stability and extrapolation difficulties when used to assess complex dynamic environments with different varieties, growth stages, and sites. It has been proven difficult to calibrate and validate some VI-based models. To address this problem, eight field experiments using eight wheat varieties were conducted during the period of 2003–2011 at four sites, and continuous wavelet transform (CWT) was applied to estimate CLB from large number of field experimental data. The analysis of 108 wavelet functions from all 15 wavelet families revealed that the best wavelet features for CLB in terms of wavelength (W) and scale (S) were observed in the near-infrared region and at high scales (7 and 8). The best wavelet-based model was derived from the Daubechies family (db), and was named db7 (W_1197_ nm, S_8_). The new model was more accurate ($R_{v}^{2}$ = 0.67 and RRMSE = 27.26%) than a model obtained using the best existing VI ($R_{v}^{2}$ = 0.54 and RRMSE = 34.71%). Furthermore, the stable performance of the optimal db7 wavelet feature was confirmed by its limited variation among the different varieties, growth stages, and sites, which confirmed the high stability of the CWT to estimate CLB with hyperspectral data. This study highlighted the potential of precision phenotyping to assess the dynamic genetics of complex traits, especially those not amenable to traditional phenotyping.

## Introduction

A key component to maintain or even increase agricultural production is therefore the development of genotyping and phenotyping technologies. Recently developed genomic approaches promise to further increase progress by breeding, while our ability to characterize the phenome of a plant has changed little. That means genomics has been advancing very rapidly, however, traditional plant phenotyping lags far behind current genotyping technique (Houle et al., 2010). This phenotyping bottleneck is of particular severity because many traits of biological and agricultural importance developed under a complex dynamic environment (Busemeyer et al., 2013). Many scientists devoted to relieve this bottleneck. They successfully developed the novel tool to represent the traditional phenotyping with low cost and high efficiency in greenhouse, for example, the high-throughput rice phenotyping facility (HRPF) (Yang et al., 2014). However, these results from controlled environment are far remove from the situation plants will experience in the field, and field conditions are notoriously hetero generous and the inability to control environmental factors makes results difficult to interpret and therefore, are difficulty to extrapolate to the filed (Araus and Cairns, 2014). Later, some novel field phenotyping systems with multi-sensor were developed for extracting the crop high-throughput phenotype properties (Bai et al., 2016; Pandey et al., 2017).

Biomass is one of the important crop phenotype traits, which are central to crop productivity. The commonly used hyperspectral vegetation index (VI) approach has been widely used to monitor crop canopy leaf biomass (CLB) at different scales due to its simplicity (Dong et al., 2003; Hese et al., 2005; Le Maire et al., 2008; Yan et al., 2013), but the non-invasive monitoring of biomass using these VIs has so far yielded only moderate prediction accuracies. For example, the VI [(ρ_NIR_/ρ_Green_) - 1], which is based on specific hyperspectral bands, can predict the green leaf biomass in corn on the ground (Gitelson et al., 2003), while the spectral reflectance (SR) index (R_900_, R_680_) can predict aboveground biomass in wheat (Serrano et al., 2000). Le Maire et al. (2008) used the normalized difference vegetation index (NDVI) (R_2160_, R_1540_) to monitor canopy foliar biomass in forests. Hansen and Schjoerring (2003) used a VI (R_708_, R_565_) to predict green biomass. These classical VIs, with a simple formulation, are very convenient in practical applications. However, hyperspectral reflectance spectroscopy has not commonly been used in plant breeding now, a major limitation to the utility of hyperspectral data is variability in environmental conditions during measurement and the spectrum feature extraction of the crop phenotype traits (Furbank and Tester, 2011). Despite the high accuracy, those VI-based models have a weak stability due to the limited number of wavebands they used. The wavebands in published hyperspectral VIs are often selected through the mathematical optimization of millions of waveband combinations that have non-casual relationships with the absorption of dry matter (Curran, 1989; Qi et al., 1995; Kokaly and Clark, 1999; Luo et al., 2013). While, the underlying mechanism of the selected waveband features is difficult to explain from the statistical results based on a limited number of samples or a poor representation of growing conditions (Noh et al., 2006; Perez-Marin et al., 2007; Atzberger et al., 2010; Yi et al., 2010). An effective way to construct a highly sensitive VI or predictive model would be to establish a comprehensive database that could represent the large variability in treatment conditions (Le Maire et al., 2008). Therefore, it is often difficult to extrapolate CLB models over different growing conditions including crop varieties, years, and ecological sites. Consequently, it is necessary to adopt a new and effective analysis method to select sensitive features from more comprehensive samples for developing more stable CLB models.

A wavelet transform (WT), as a new emerging signal processing method, has been recently used to characterize the spectral information in the crop classification, estimation of forest leaf area index (LAI) and ^∗∗^canopy closure (CC) structural parameters, and the determination of leaf mass per unit area, plant leaf water content, and chlorophyll content (Koger et al., 2003; Pu and Gong, 2004; Cheng et al., 2010, 2011, 2014). Some previous studies (Koger et al., 2003; Pu and Gong, 2004) have focused on a discrete wavelet transform (DWT); however, the continuous wavelet transform (CWT) method could be accurately used to extract spectral features (Cheng et al., 2010, 2011, 2014). To date, the potential to undertake a wavelet analysis of hyperspectral reflectance with CWT for the crop biomass model has not been well documented. In addition, little is known regarding the selection of appropriate spectral features using the CWT method for crop biomass. Most existing studies have selected the wavelet function and wavelet features based on waveform similarity (Blackburn and Ferwerda, 2008; Bigerelle et al., 2013) or have focused directly on the most commonly used Mexican Hat wavelet function (Muraki, 1995; Torrence and Compo, 1998; Cheng et al., 2010, 2011, 2014). Whether the Mexican Hat is the best function for extracting hyperspectral information for crop biomass remains unknown.

Here, 108 wavelet functions from 15 wavelet families were investigated to select the sensitive wavelet features for CLB, which were used to develop robust models with hyperspectral data. These data were collected under different nitrogen rates, planting densities, and varieties of winter wheat in eight field experiments over 8 years at four sites. Such a comprehensive dataset has the potential to produce a reliable model for the spectroscopic estimation of CLB. To evaluate the performance of the new model, we compared the accuracy and stability of the prediction with the existing VI models under various growing conditions with different partitioning strategies.

## Materials and Methods

### Experimental Design

Eight field experiments were conducted for eight consecutive years in this study. The experiment involved various nitrogen levels and density measurements, and included eight varieties of wheat grown at four sites. A randomized complete-block design was used with three replications per plot. For all treatments, sufficient Ca(H_2_PO4)_2_ and KCl were applied (150 kg ha^-1^) prior to seeding. Crop management followed local standard practices for wheat production. The detailed experimental design is described in **Table 1**.

**Table 1:** Summary of the conditions used in the eight wheat growth experiments.

Exp.	Site	Sowing time	Nitrogen rate (kg ha^-1^)	Variety	Sampling dates	No. of samples	Function
1	JAAS	November 2, 2003	0, 75, 150, 225, 300	Ningmai 9 Huaimai 20 Yangmai 10 Xumai 26	April 8, April 20, May 4, May 17	127	Validation
2	JAAS	October 31, 2004	0, 75, 150, 225, 300	Ningmai 9 Yumai 34 Yangmai 10	March 19, April 13, April 23, May 6, May 19, May 25	219	Calibration
3	NAFB	November 3, 2005	0, 90, 180, 270	Ningmai 9 Yumai 34	March 30, April 11, April 20, April 29, May 19, May 24, June 2	136	Validation
4	JPF, NJAU	November 1, 2007	0, 90, 180, 270	Ningmai 9	March 11, March 25, April 18, April 25, May 6, May 20	126	Calibration
5	YCY	November 6, 2009	150, 225	Yangmai 16 Aikang 58	March 11, March 19, March 28, April 19, April 28, May 3, May 11, May 20	285	Calibration
6	YCY	November 5, 2009	0, 90, 180, 270	Yangmai 16	March 10, March 19, April 9, April 15	203	Validation
7	YCY	November 10, 2009	0, 75, 150, 225, 300	Ningmai 13 Yangmai 16	March 12, March 18, March 28, April 8, April 16, April 30	240	Calibration
8	YCY	November 9, 2010	150, 225	Yangmai 16	February 23, March 4, March 10, March 23, March 30, April 10, April 19, April 25, May 1, May 13, May 18	166	Calibration


**The number after the variety indicates the time of breeding for the variety.* JAAS, Jiangsu Academy of Agricultural Sciences (N32.03°, E118.87°); NAFB, Nanjing Agricultural and Forestry Bureau (N32.05°, E118.79°); JPF, NJAU, Jiangpu farm, Nanjing Agricultural University (N32.03°, E118.03°); and YCY, Yizheng county in Yangzhou (N32.32°, E119.30°).*

### Measurements

#### Canopy Leaf Biomass

After each measurement of canopy SR, an area of 0.25 m^2^ (two rows × 0.5 m long) of wheat plants from each plot was collected for the determination of leaf dry biomass per unit ground area at the canopy scale (CLB, g DW m^-2^). For each sample, all green leaves were separated from the stems, oven-dried at 70°C to a constant weight, and then weighed.

Among the eight experiments, the average CLB values ranged from 0.0096 to 0.330 kg/m^2^ with the lowest mean CLB being in EXP.5 and the highest in EXP.2. All standard deviations were less than 0.0650 kg/m^2^, and the variance of the CLB in intergroup experiments was less than 0.05 kg/m^2^ (**Table 2**).

**Table 2:** Basic canopy leaf biomass (CLB) statistics for the eight experimental data sets (kg/m^2^).

Exp.	Min.	Max.	Mean	Std. Dev.	No. of samples
5	0.0096	0.240	0.088	0.0450	285
6	0.0154	0.190	0.098	0.0387	203
8	0.0184	0.301	0.105	0.0565	166
7	0.0185	0.298	0.107	0.0617	240
3	0.0278	0.290	0.109	0.0530	136
4	0.0349	0.218	0.113	0.0507	126
1	0.0537	0.330	0.164	0.0650	127
2	0.0507	0.327	0.183	0.0566	219


#### Canopy Hyperspectral Reflectance

All canopy hyperspectral measurements were made using an ASD FieldSpec Pro spectrometer (Analytical Spectral Devices, Boulder, CO, United States). This spectrometer is fitted with 25° field of view fiber optics operating in the 350–2500 nm spectral region with a sampling interval of 1.4 nm and spectral resolution of 3 nm between 350 and 1050 nm, and 2 and 10 nm, respectively, between 1050 and 2500 nm. The measurements were conducted at a height of 1.0 m above the vertical canopy (the height of wheat was 75–90 cm at maturity) and with a 0.44 m view diameter under clear sky conditions between 10:00 and 14:00 (Beijing local time).

Measurements of reflectance values were acquired at 10 sampling sites in each plot, with each sample observation averaging 20 scans at the optimized integration time. The resulting spectrum file contained a continuous SR at 1 nm steps over the band region of 350–2500 nm. A white panel SR value was taken before and after the vegetation measurement, with two scans obtained each time. In each experiment, data were obtained at several major growth stages, as detailed in the description of the experimental method. The spectral regions 1350–1410, 1790–1950, and 2471–2500 nm were excluded from the spectral analysis due to the strong absorption of water in the atmosphere. Because the CWT method requires a continuous spectrum, the reflectance values in the above three regions were set to zero in this study to avoid interference from noise.

### Continuous Wavelet Decomposition of Canopy Hyperspectral Spectra

#### Continuous Wavelet Transform (CWT)

Wavelets are mathematical functions that are used to dissect data into different frequency components, with each component having a resolution appropriate to its scale (Grinsted et al., 2004). It is a gradual multiscale refinement of the signal (function) through an expansion and shift operation (Mallat, 1989). A wavelet transform can be a discrete wavelet transform (DWT) or continuous wavelets transform (CWT). DWT can reduce the redundant information within a transformation, but it may miss useful signal information, and the superiority of continuous over discontinuous decomposition is possibly due to the greater amount of spectral detail (variation with wavelength) (Blackburn and Ferwerda, 2008). Hence, we used the CWT method in this study. Equation (1) is the functional formula of the wavelet coefficient of the CWT:

(1) $$C(a,b;f(t),\varphi \left(t\right))=\int_{-\infty }^{+\infty }f(t)\frac{1}{\sqrt{a}}\varphi *(\frac{t-b}{a})dt$$

In this study, C is the wavelet coefficient after transformation that expresses the similarity between the original spectra and the wavelet function under a specific scaling and translation; a is the scaling factor (scale), we selected a power (a = 3, 4, 5…, 8, which represents 2^3^, 2^4^, 2^5^…, 2^8^); *b* is the shifting factor (displacement); *f*(*t*) represents the SR of each wavelength; φ(*t*) is the wavelet basis function (wavelet mother function), depending on the parameters of ^a^ and ^b^, that contains 108 wavelet functions from 15 wavelet families (**Table 3**); and t represents each wavelength (t = 350, 351, 352…, 2,500 nm). All of the wavelet coefficients were used to estimate the CLB.

**Table 3:** List of 15 wavelet families encompassing 108 wavelet functions used in this study.

Name of the wavelet family	Short name	Wavelet function in the family	Number of wavelet function	Reference
1. Haar	Haar	Haar	1	Daubechies, 1992
2. Daubechies	Db	db2, db3, …, dbN, …, db20	19	
3. Symlets	Sym	sym2, sym3, …, symN, …, sym17	16	
4. Coiflets	Coif	coif1, coif2, …, coifN, …, coif5	5	
5. Biorthogonal	Bior	bior Nr.Nd (Nr = 1, Nd = 1, 3, 5; Nr = 2, Nd = 2, 4,6,8; Nr = 3, Nd = 1, 3, 5, 7, 9; Nr = 4, Nd = 4; Nr = 5, Nd = 5; Nr = 6, Nd = 8)	15	
6. Reverse Bior	Rbio	rbio Nr.Nd (Nr = 1, Nd = 1, 3, 5; Nr = 2, Nd = 2, 4,6,8; Nr = 3, Nd = 1, 3, 5, 7, 9; Nr = 4, Nd = 4; Nr = 5, Nd = 5; Nr = 6, Nd = 8)	15	
7. Meyer	Meyer	Meyr	1	
8. Mexican hat	Mexh	Mexh	1	
9. Morlet	Morl	Morl	1	
10. Complex Gauusian	Cgau	cgau1, cgau2, …, cgauN, …, cgau8	8	
11. Complex Shannon	Shan	shan Fb-Fc (Fb = 1, Fc = 0.1, 0.5, 1, 1.5; Fb = 2, Fc = 3)	5	Teolis, 1998
12. Complex Frequency B-Spline	Fbsp	fbsp M-Fb-Fc (M = 1, Fb = 1, Fc = 0.5, 1, 1.5; M = 2, Fb = 1, Fc = 0.1, 0.5, 1)	5	
13. Complex Morlet	Cmor	cmor Fb-Fc (Fb = 1, Fc = 0.4, 0.5, 1, 1.5; Fb = 2, Fc = 0.1, 0.5)	5	
14. Dmeyer	Dmey	Dmey	1	Addison, 2002
15. Gaussian	Gaus	gaus1, gaus2, …, gausN, …, gaus8	8	Bernardino and Santos-Victor, 2005


#### Determination of the Optimal Wavelet Function, Wavelength, and Scale

**Figure 2** displays the procedure for determining the optimal wavelet function, the best wavelength, and scale. First, the wavelet function was selected, and the CWT then proceeded to obtain the wavelet coefficient. A linear regression model was then established between the transformed wavelet coefficient and the biomass. The top 1% of coefficient of determination ($R_{c}^{2}$) values were determined, The model was validated with the dependent data, and we then defined the sensitive wavelength and scale for the top 1% of $R_{c}^{2}$ and $R_{v}^{2}$ values ($R_{c}^{2}$ and $R_{v}^{2}$ are the coefficient of determination for calibration and validation, respectively), and the relative root mean square error (RRMSE). We repeated this procedure for all 108 wavelet functions from the 15 families. After systematically comparing the performance of the calibration and validation, the best wavelet mother function was determined using a box plot. A box plot was used in this study to select the optimal wavelet function, in which the outliers can describe the optimal and the poor wavelet functions (Grubbs, 1969; Pierce and Chick, 2013).

**FIGURE 1:**
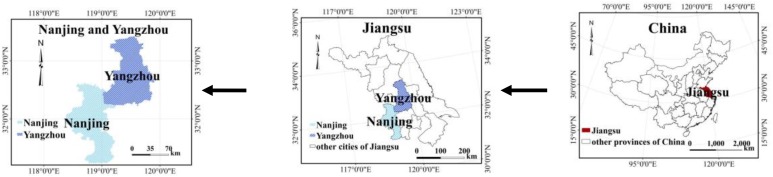
The study site and the location of the experiments.

**FIGURE 2:**
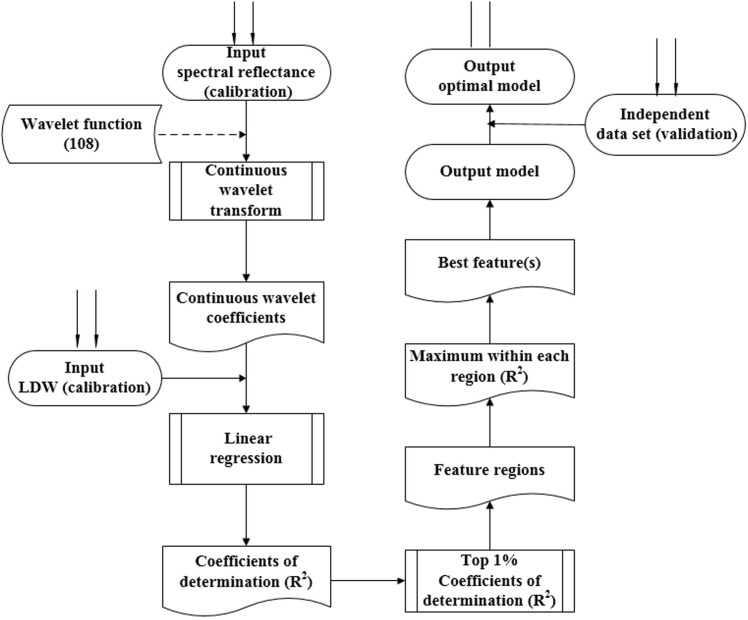
The procedure used for determining the optimum wavelet function, sensitive wavelength, and best scale. The dashed lines represent the same analysis of 108 wavelet functions from 15 wavelet families.

### Calibration and Validation of the Model

Calibration data from experiments 2, 4, 5, 7, and 8, including information obtained from the various varieties, growth stages, and sites, were selected to construct the CLB model, while data from the remaining three experiments (1, 3, and 6) were used to test the constructed model equation. This partitioning strategy ensured a good representation of the entire data set with calibration samples and an approximate ratio of 2:1 for the number of calibration (*n* = 1036) and validation (*n* = 466) samples.

In addition to $R_{c}^{2}$ and $R_{v}^{2}$, the relative root mean square error (RRMSE) and stand error (SE) were used to evaluate the fit between the predicted and observed data along with a 1:1 plotting of the two sets of values. The RRMSE was calculated using the following equation (Yao et al., 2010):

(2) $$RRMSE=\sqrt{\frac{1}{n}\times \overset{n}{\underset{i=1}{\Sigma }}{\left(P_{i}-O_{i}\right)}^{2}\times \frac{100\%}{\overset{¯}{O_{i}}}}$$

where, *P*_i_ is the predicted biomass value of the model, *O_i_* is the biomass value of an observation, *n* is the sample number, and $\overset{¯}{O_{i}}$ the mean of the validation biomass data. All of these procedures were completed with self-programmed software based on MATLAB 8.1 (The MathWorks, 2013) and SPSS 20.0 software.

## Results

### Selection of the Optimal Wavelet Function

**FIGURE 3:**
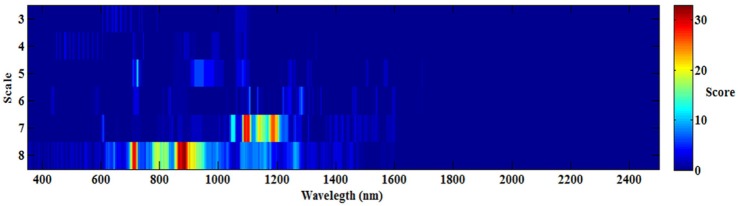
Occurrence score of all the wavelet functions under various wavelengths and scales.

To determine the best wavelet features [wavelength (W) and scale (S)] for all 108 wavelet functions, the occurrence score for every feature (W and S) was counted and is shown in **Figure 3**. The maximum score was 33, while the minimum score was nearly 0, which indicated that not all the wavelet functions had the same features (W and S), and different wavelet functions may have special features for each optimum model. The best features were mostly frequently observed at the scale of 7 and 8, and the sensitive wavelength was found to be located in the near-infrared region (780–1,350 nm). Therefore, when estimating CLB based on CWT, a near-infrared wavelength (780–1,350 nm) and at scale of 7 or 8 would be most effective.

**FIGURE 4:**
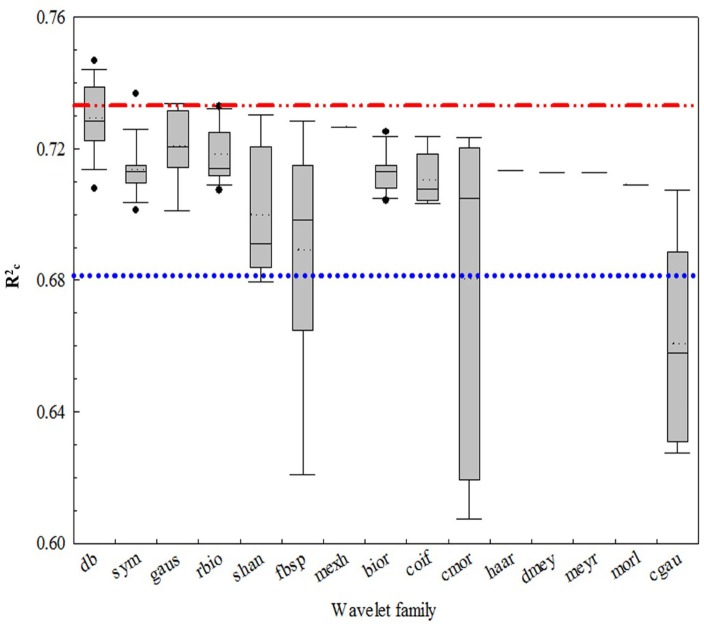
The coefficient of determination of calibration ($R_{c}^{2}$) values for the canopy leaf biomass (CLB) models for 15 wavelet families selected using a box plot. The blue and red lines are the interval lines of the general performance of the wavelet function. The region above the red line is the optimum wavelet function and the region below the blue line is the area of poor wavelet function.

**Figure 4** shows that all of the coefficients of determination ($R_{c}^{2}$) for the CLB models from the 15 wavelet families were over 0.62 with the Daubechies (db) families being the highest ($R_{c}^{2}$ from 0.707 to 0.747). Among the 108 wavelet functions, the best functions were db7, db16, db17, db8, db6, db20, sym3, db3, gaus3, and db4, and the worst were shan1-1, fbsp1-1-1.5, fbsp2-1-1, cmor1-1, cmor-1.5, cgau4, cgau5, cgau6, cgau7, and cgau8. The wavelet function with the highest *R*^2^ value was db7 ($R_{c}^{2}$ = 0.75 and SE = 0.032 kg/m^2^), while the commonly used mexh was 0.73 and 0.034 kg/m^2^, respectively.

### Determination of the Optimal Wavelet Features and Models

**FIGURE 5:**
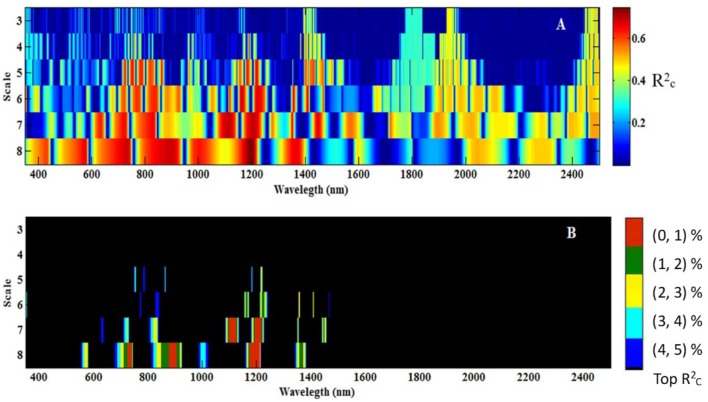
Correlation scalograms for the coefficient of determination ($R_{c}^{2}$) of the canopy leaf biomass (CLB) model under db7 with variable wavelength and scale (A), and the top 1% to top 5% of $R_{c}^{2}$ values (B). The scale (3, 4, 5…, and 8) in **Figure 5** represents 2^3^, 2^4^, 2^5^…, and 2^8^.

All the data for db7 were processed with CWT. **Figure 5A** shows the correlation scalogram for the linear regression between the CLB and db wavelet coefficient at the scale from 3 to 8 and at wavelengths from 350 to 2500 nm. The top 1–5% of $R_{c}^{2}$ values were extracted and are shown in **Figure 5B**. Five featured regions (1,099–1122 and 1,194–1,216 nm at scale 7; 724–738, 878–905, and 1,173–1,210 nm at scale 8) were identified using the calibration data, which were identified at higher scales (7 or 8) and in the near-infrared region, except for the range of 724–738 nm (**Figure 5B**, top 1%).

In this study, the wavelet feature was determined according to the $R_{c}^{2}$ value and the absorption principle. Therefore, five wavelet features [(W_732_, S_8_), (W_894_, S_8_), (W_1111_, S_7_), (W_1197_, S_8_), and (W_1206_, S_7_)] were selected with db7 (W_1197_, S_8_) being the optimum due to it having the highest $R_{c}^{2}$ and db7 (W_894_, S_8_) being the worst (**Table 4**).

**Table 4:** Assessment of the five best db7 wavelet features in the estimation of canopy leaf biomass (CLB).

Wavelet Feature	Equation	Calibration (*N* = 1036)		Validation (*N* = 466)	
			
		$R_{c}^{2}$	SE (kg/m^2^)	$R_{v}^{2}$	RRMSE (%)
db7 (W_1197_, S_8_)	Y = 0.428x+0.070	0.75	0.032	0.67	27.26
db7 (W_1206_, S_7_)	Y = 0.375x-0.002	0.69	0.036	0.68	23.86
db7 (W_1111_, S_7_)	Y = -0.362x+0.033	0.69	0.036	0.68	23.13
db7 (W_732_, S_8_)	Y = 0.292x+0.009	0.69	0.036	0.73	20.79
db7 (W_894_, S_8_)	Y = -0.199x+0.017	0.68	0.036	0.72	20.91


**FIGURE 6:**
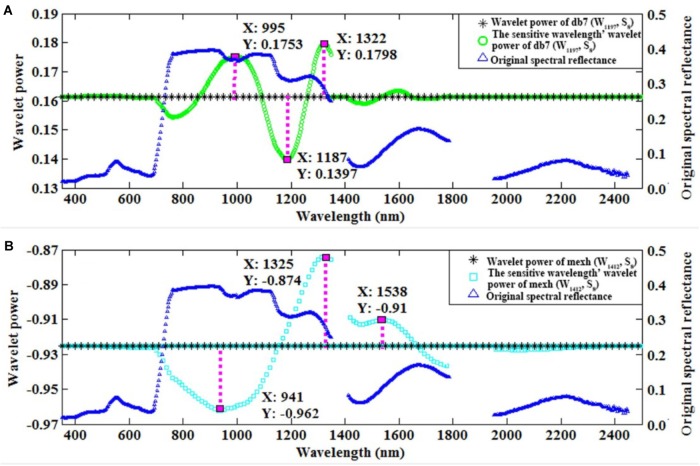
The original spectral reflectance, wavelet power, and sensitive wavelength’ wavelet power of db7 (W_1197_, S_8_) **(A)** and mexh (W_1412_, S_8_) **(B)**.

Furthermore, we used the control variable method to qualitatively analyze which wavebands affected the wavelet feature for db7 (W_1197_, S_8_) and the commonly used mexh (W_1412_, S_8_). The convolution algorithm was used to transform the original spectrum into the wavelet power on a specific wavelet function. Therefore, the wavelet power would be influenced by the wavelength, shape, and scale of a certain wavelet function. As consequence, the wavelet feature could be impacted by the neighboring region of the characteristic wavelength. We found that the features of db7 (W_1197_, S_8_) were mainly affected by the wavelengths of 995, 1187, and 1322 nm (**Figure 6A**). The features of mexh (W_1412_, S_8_) were mainly affected by the wavelengths of 941, 1325, and 1538 nm (**Figure 6B**).

**FIGURE 7:**
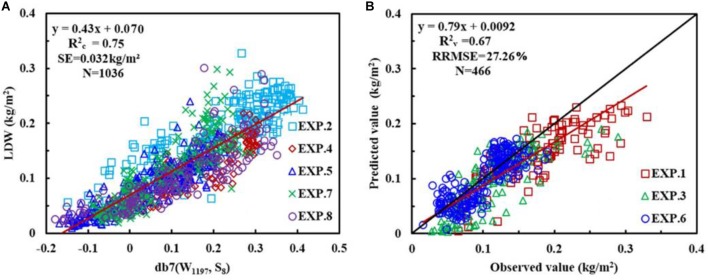
The relationship between canopy leaf biomass (CLB) and wavelet power for db7 (W_1197_, S_8_) **(A)**, and the 1:1 relationship between the predicted and observed CLB for db7 (W_1197_, S_8_) **(B)**.

Considering the $R_{c}^{2}$, SE, and the sensitive wavelength, the db family with a wavelength of 1197 nm and at scale of 8, i.e., db7 (W_1197_, S_8_), was determined to be the optimal wavelet function and feature for constructing the CLB model, and calibration and validation of db7 (W_1197_, S_8_) are shown in **Figure 7**.

### Comparison of the CLB Models With Previous Models

To determine whether the new model was comparable to previously reported CLB models for wheat, the data collected in this present study were applied to compare the performance of the model for db7 (W_1197_, S_8_) with its performance for mexh (W_1412_, S_8_), NDVI_Bleaf_, and RVI_GBM_ (**Table 5**).

**Table 5:** Comparison of the model performance for db7 (W_1197_, S_8_) with its performance for the commonly used mexh (W_1412_, S_8_), NDVI_Bleaf_, and RVI_GBM_.

Spectral feature		Calibration (*N* = 1036)		Validation (*N* = 466)		Reference
				
		$R_{c}^{2}$	SE (kg/m^2^)	$R_{v}^{2}$	RRMSE (%)	
**Wavelet feature**					
db7	(W_1197_, S_8_)	0.75	0.032	0.67	27.26	This paper
Mexh	(W_1412_, S_8_)	0.73	0.034	0.68	23.63	This paper
**Spectral index**					
NDVI_Bleaf_	(R_2160_, R_1540_)	0.62	0.040	0.54	34.71	Le Maire et al., 2008
RVI_GBM_	(R_708_, R_565_)	0.50	0.045	0.36	34.38	Qi et al., 1995


The results showed that the best model was db7 (W_1197_, S_8_), which had a high accuracy and low predictive performance ($R_{c}^{2}$ = 0.75, $R_{v}^{2}$ = 0.67 and RRMSE = 27.26%), that was slightly higher than that of the commonly used mexh function (W_1412_, S_8_; $R_{c}^{2}$ = 0.73, $R_{v}^{2}$ = 0.68 and RRMSE = 23.63%). It also had a better performance than the existing indices NDVI_Bleaf_ ($R_{c}^{2}$ = 0.62, $R_{v}^{2}$ = 0.54 and RRMSE = 34.71%) and VI_GBM_ ($R_{c}^{2}$ = 0.50, $R_{v}^{2}$ = 0.36 and RRMSE = 34.38%) (**Table 5**).

## Discussion

### Stability and Extrapolation of the New CLB Model

In this study, 1502 comprehensive samples were used to compare the stability and extrapolation of a newly developed CLB model with an existing VI model (**Table 6**). The abundance of experimental data enabled an authoritative assessment of the CLB model performance to be made. We categorized the samples into four sub-groups (growth stages, sites, varieties, and years). In the validation of the new model, the $R_{v}^{2}$ value was higher than that of the existing VIs, while the RRMSE was lower than that of the VI-derived models. This indicates that the new model based on db7 (W_1197_, S_8_) was very stable and could be effectively extrapolated across a diverse range of growth stages, sites, varieties, and years. The results were consistent with those of Cheng et al. (2014), who also found that the transferability of the wavelet-based predictive model to the entire measured database was either better than or comparable to a VI-derived model.

**Table 6:** Comparison of the stability and extrapolation potential for models based on db7 and NDVI_Bleaf_ when categorizing samples using the growth stage, site^∗^, variety, and year.

Grouping variable	Sub-group	Validation				
			
		Sample number	Db7 NDVI_Bleaf_			NDVIBleaf
				
			$R_{v}^{2}$	RRMSE (%)	$R_{v}^{2}$	RRMSE (%)
Anthesis	After	215	0.57	30.42	0.44	38.24
	Before	251	0.76	23.51	0.63	31.46
	Mean		0.66	26.97	0.53	34.85
Site	Site 1	127	0.68	17.84	0.51	21.80
	Site 2	136	0.64	34.77	0.45	45.64
	Site 4	203	0.67	25.35	0.70	27.50
	Mean		0.67	25.99	0.55	31.64
Variety	Huaimai 20	41	0.60	21.81	0.52	22.70
	Ningmai 9	108	0.74	25.43	0.54	35.66
	Xumai 26	28	0.70	14.40	0.53	21.87
	Yangmai 10	18	0.80	13.51	0.53	13.19
	Yumai 34	68	0.67	36.40	0.50	48.24
	Yangmai 16	203	0.67	25.35	0.70	27.50
	Mean		0.70	22.82	0.55	28.19
Year	2003–2004	127	0.68	17.84	0.51	21.80
	2005–2006	136	0.64	34.77	0.45	45.64
	2009–2010	203	0.67	25.35	0.70	27.50
	Mean		0.67	25.99	0.55	31.64
Total	Mean		0.67	25.44	0.55	31.58


*^∗^Site 1: JAAS; Site 2: NAFB; Site 4: YCY.*

In addition, we noticed that the performance of the new model before anthesis was much better than in the stage after anthesis. The reason for this may be that the canopy cover in the later growth stage (after anthesis) was affected by panicle anthesis or grain development, which may increase the background noise. The model had a similar accuracy among sites and years and therefore could be extrapolated to different sites or years. However, there was less stability among the varieties and we therefore speculated that the plant type or structure among the different varieties influenced the reflectance. It should be considered how to reduce the impact of this issue in the future studies.

### The Reason for the Higher Stability and Extrapolation Potential

#### Sensitive Wavelengths for Monitoring CLB

Previous studies have constructed the following VIs: NDVI (R_2160_, R_1540_) (Le Maire et al., 2008), [(ρ_NIR_ / ρ_Green_)-1] (Gitelson et al., 2003), SR (R_900_, R_680_) (Serrano et al., 2000), and VI_GBM_ (R_708_, R_565_) (Qi et al., 1995) to predict crop biomass. However, the sensitive wavebands of 565, 680, 708, 900, 1540, and 2160 nm are not the core wavelengths of absorption, with the exception of 900 nm (the absorption band of protein) and 1540 nm (the absorption band of starch and cellulose) (Curran, 1989). In this study, the results show that db7 (W_1197_, S_8_) was mainly affected by the wavelengths of 995, 1187, and 1322 nm. The wavelength of 995 nm induces bending of the O-H bond and is the absorption peak of starch (Curran, 1989). In addition, 1187 and 1197 nm are close to 1200 nm, which is the absorption wavelength of water, cellulose, starch, and lignin (Curran, 1989; Qi et al., 1995; Bernardino and Santos-Victor, 2005; Cheng et al., 2014). Each of 1187, 1197, and 1322 nm are located in the near-infrared region, which is sensitive to the LAI (Pu and Gong, 2000). Therefore, db7 (W_1197_, S_8_) could provide information about LAI and LB. Fortunately, CLB is equal to the product of LAI and LB. Therefore, it is sensible to include the sensitive regions of both LAI and LB when selecting the sensitive region of CLB.

**FIGURE 8:**
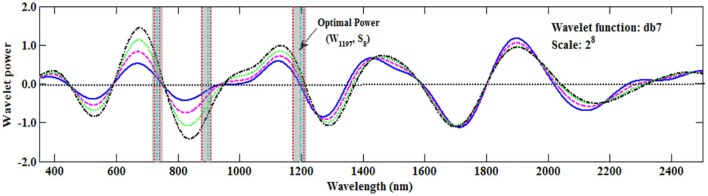
The wavelet coefficients of the Db function on S_8_. The areas between the two red vertical lines (the transparent shadow) represent the top 1% of $R_{c}^{2}$ values in **Figure 5B**, and the green vertical lines represent the maximum $R_{c}^{2}$ value at the band position (**Table 4**).

Most studies have used the sensitive wavelength at which the wavelet coefficient is at a maximum (peak) or minimum (valley) (Koger et al., 2003). However, in this study the sensitive wavelength region at the zero value of the wavelet coefficient was found to be better than that of the peak or valley (**Figure 8**), which was similar to the result reported by Cheng et al. (2010). This would provide a new method to determine the sensitive wavelength. Therefore, in the future we should pay more attention to those wavelengths in which the wavelet coefficients are close to zero.

#### Optimal Wavelet Function and Scales

Previous studies have commonly used the function of mexh, db, haar, and bior to extract spectral features for mapping forest LAI and CC (Pu and Gong, 2004), detecting insect damage (Cheng et al., 2010), and plant leaf water content (Cheng et al., 2011). In addition, they implied that predictive accuracy varied according to the choice of wavelet function in some cases; hence, a judicious choice of wavelet function may be necessary. Our study confirmed this viewpoint. **Figure 4** shows that the choice of mother wavelet could greatly affect the efficacy of the wavelet-based feature when changing the mother wavelet. Blackburn and Ferwerda (2008) selected the optimal wavelet function, and found that bior 1.3 and rbior 5.5 at scale 6 were the best wavelet features when estimating chlorophyll concentration using a CWT and DWT. However, they only focused on 53 individual wavelet functions at the scale of 0–8. In this study, the best wavelet function and features for CLB were determined based on the 108 individual wavelet functions at the scale of 2^3^–2^8^.

Koger et al. (2003) set an accuracy threshold to select the optimal mother wavelet function, and found that only Haar, db5, db10, bior 2.2, bior 2.4, bior 2.6, bior 2.8, bior 6.8, sym2, and sym7 could qualify the accuracy, with the very simple Haar mother wavelet being the best. However, in our study we determined that the db7 families produced the best performance, which differed from previous results. Therefore, the mother wavelet function should first be applied when the CWA method is used, and the application of commonly used mother wavelet functions should not be taken for granted.

The analysis in this study incorporated many wavelet functions. There were some similar performances between the wavelets within each family, which may account for the lack of a significant statistical difference. This has commonly been the case in previous research. Therefore, it may be possible to develop a new wavelet function specifically for this application, and there are precedents for this approach (e.g., the MATLAB wavelet toolbox) (Blackburn and Ferwerda, 2008). In addition, it may be useful to decompose spectra using a group of wavelet functions that perform well overall, deriving a series of predictive regression models and obtaining an average value of the estimated parameters.

## Conclusion

A comprehensive analysis of 108 individual wavelet functions from all 15 wavelet families revealed that the best wavelet features for the CLB were mostly located in the near-infrared region and at high scale (7 or 8). The best wavelet-based model was derived from the db family and was named db7 (W_1197_ nm, S_8_). It was affected by the neighboring wavelengths of 995, 1187, and 1322 nm. The new model had a better accuracy than that obtained using the existing VIs. Furthermore, the more stable performance with the db7 wavelet feature was further confirmed by the lack of variation in the $R_{c}^{2}$ and $R_{v}^{2}$ values across different varieties, growth stages, sites, and years. The main outcomes of this study were: (1) the use of 108 CWT methods to select the wavelet function for crop biomass from the hyperspectral data, (2) the identification of the wavelengths in the zero 38 region of the wavelet coefficient, (3) the development of a more stable and robust crop biomass model based on CWT, and (4) the resolution of some of the problems associated with the existing methods. The use of the CWT method would provide theoretical and technical support for the monitoring of crop growth parameters.

However, we only tested the one-dimensional wavelet decomposition functions available in the MATLAB package due to the limited amount of original data. In the future, two-dimensional WTs should be studied to assess the suitability of hyperspectral image data for extracting spatial information (such as texture and shape). In addition, it should be determined whether a CWT or DWT is a more accurate biomass estimation method. Moreover, whether the same structure or physical parameter would have a similar wavelet function and features for vegetation should also be checked.

## Author Contributions

YZ conceived the study. XY and HS also conceived the study, analyzed the data, and wrote the manuscript. HS and MJ finished the laboratory experiment. TC, MJ, YT, QC, WC, CC, JC, and RG proofread and made comments on the manuscript. All authors participated in the review process.

## Conflict of Interest Statement

The authors declare that the research was conducted in the absence of any commercial or financial relationships that could be construed as a potential conflict of interest.
